# Assessment of COVID-19 Vaccine Effectiveness Against SARS-CoV-2 Infection, Hospitalization and Death in Mexican Patients with Metabolic Syndrome from Northeast Mexico: A Multicenter Study

**DOI:** 10.3390/vaccines13030244

**Published:** 2025-02-27

**Authors:** Beatriz Silva Ramírez, Katia Peñuelas Urquides, Brenda Leticia Escobedo Guajardo, Viviana Leticia Mata Tijerina, Jorge Eleazar Cruz Luna, Roberto Corrales Pérez, Salvador Gómez García, Laura Adiene González Escalante, María Elena Camacho Moll

**Affiliations:** 1 Laboratory of Immunogenetics, Northeast Biomedical Research Center, Mexican Social Security Institute, Monterrey 64720, Nuevo Leon, Mexico; silbear2002@yahoo.es (B.S.R.); vivianamata@gmail.com (V.L.M.T.); 2 Laboratory of Molecular Microbiology, Northeast Biomedical Research Center, Mexican Social Security Institute, Monterrey 64720, Nuevo Leon, Mexico; katia.penuelasu@imss.gob.mx (K.P.U.); lgonzalezescalante@gmail.com (L.A.G.E.); 3 Laboratory of Molecular Research of Diseases, Northeast Biomedical Research Center, Mexican Social Security Institute, Monterrey 64720, Nuevo Leon, Mexico; leticiaescobedo@hotmail.com; 4 Medical Epidemiological Assistance Coordination of the State of Nuevo Leon, Mexican Social Security Institute, Monterrey 64000, Nuevo Leon, Mexico; jorge.cruzlu@imss.gob.mx (J.E.C.L.); roberto.corrales@imss.gob.mx (R.C.P.); salvatoregomezg@hotmail.com (S.G.G.); 5 Laboratory of Molecular Biology, Northeast Biomedical Research Center, Mexican Social Security Institute, Monterrey 64720, Nuevo Leon, Mexico

**Keywords:** metabolic syndrome, COVID-19 vaccines, vaccine efficacy, SARS-CoV-2 variants

## Abstract

**Background/Objectives:** Metabolic syndrome (MetS) is a predisposing factor for severe COVID-19. The effectiveness of COVID-19 vaccines in patients with MetS has been poorly investigated. The aim of this study was to evaluate the effectiveness of COVID-19 vaccination before (BO) and after the Omicron (AO) SARS-CoV-2 variant in patients with MetS. **Methods:** This retrospective observational study was carried out in a total of 3194 patients with MetS and a COVID-19 PCR or rapid antigen test. The main outcomes were vaccine effectiveness against infection, hospitalization and death resulting from COVID-19. **Results:** BO, only two doses of BNT162b2 were effective against infection, this effectiveness was lost AO. Also, with two doses, BNT162b2, ChAdOx1 and CoronaVac were effective against hospitalization BO; however, AO, only BNT162b2 and CoronaVac were effective. Regarding death as an outcome of COVID-19, two doses of BNT162b2 were effective BO, whereas AO, BNT162b2 and CoronaVac were 100% effective. BO the presentation of a sore throat increased after two doses of COVID-19 vaccine regardless of the type, and the presentation of dyspnea diminished after two doses of BNT162b2 and CoronaVac. **Conclusions:** The SARS-CoV-2 Omicron variant has impacted vaccines’ effectiveness against hospitalization and death in patients with MetS. A tailored vaccination scheme for patients with MetS should be implemented due to the varying effectiveness rates observed in our study.

## 1. Introduction

After the initial outbreak in December 2019 and quickly spreading throughout the world, COVID-19 became a pandemic by March 2020 and has since been evolving. Although the severity of subsequent waves was mitigated by the introduction of various vaccines, their effectiveness depends largely on distribution within populations. Moreover, recently identified mutant strains have shown different levels of virulence and vaccine resistance [1,2]. To prevent the spread of the disease, it is crucial to identify groups at higher risk of infection, particularly in cases of suspected exposure to SARS-CoV-2 and during periods of active viral replication [3,4].

Several studies have demonstrated a lowered COVID-19 vaccine effectiveness in patients with comorbidities including cancer [5], hypertension [6], obesity [6], chronic kidney disease [7], and other conditions [8].

Diabetes, one of the components of the metabolic syndrome (MetS) and one of the most common comorbidities present in COVID-19-positive patients, is associated with a significantly increased risk of intensive care unit (ICU) admission and mortality [9,10]. Another component of MetS is hypertension, and the impact of hypertension on COVID-19 severity is under debate as other factors are associated with hypertension such as older age and cardiovascular risk factors, which might increase COVID-19 infection and progression [11]. Finally, the third component is obesity, which is a major risk factor for severe COVID-19 [12]. While vaccines have proven effective in preventing COVID-19-related hospitalization and mortality, information regarding their effectiveness in patients with comorbidities, particularly before and after the emergence of Omicron, is still being explored [6,13,14,15,16,17,18,19,20,21,22,23,24,25,26,27,28,29]. Research regarding vaccine effectiveness in patients with and without MetS is limited [30,31]. Metabolic syndrome is a condition in which vaccine response might be hampered by the cytokine storm caused by the excess of adipose tissue, which ultimately dysregulated innate immune cells in the lungs. Such dysregulation can lead to the overproduction of inflammatory cytokines and chemokines by immune cells, which in turn attract additional inflammatory mediators [32,33]. A sexual and ethnic component has also been described in the development of MetS, for instance, in men, there is a predisposition to meta-inflammation [34], and MetS is notably more prevalent in men than women [35,36]. Regarding the ethnic component, studies have shown that there is a higher prevalence of MetS among Americans and Europeans compared to Asians [37]. This disparity may be due to COVID-19 severity, hospitalization, and mortality rates among different countries.

In Mexico, the prevalence of MetS has alarmingly increased in recent decades with a 40.2%, 57.3%, 59.99%, and 56.31% prevalence in 2006, 2012, 2016 and 2018, respectively, with the research in [38] underscoring the urgent need to address this public health matter.

COVID-19 vaccination in Mexico was performed in stages. Stage one was carried out from December 2020 to February 2021, prioritizing frontline health care workers, followed by a second stage (February–April 2021) where the rest of the frontline health care workers and people aged 60 and older were vaccinated. During the third stage (April–May 2021), schoolteachers and people aged 50–59 were vaccinated. During the fourth stage (May–June 2021), people aged 40–49 were vaccinated and the final stage (June 2021 to present) included the rest of the population [39]. The type of vaccines administered was availability dependent. Among the mainly used vaccines there were BNT162b2 (Pfizer-BioNTech), ChAdOx1 (AstraZeneca), CoronaVac (Sinovac Life Sciences), Gam-COVID-Vac (Gamaleya’s Sputnik V), mRNA-1273 (Moderna), NVX-CoV2373 (Novavax) and BBIBP-CorV (Sinopharm).

In Mexico, the COVID-19 pandemic unfolded in multiple waves, driven by the emergence of successive variants, which hindered the impact of mass vaccination campaigns. This study categorizes data into two periods: pre-Omicron and post-Omicron. Evidence suggests that the efficacy of vaccines against Omicron infection is significantly lower compared to earlier variants such as Alpha, Beta, Gamma, and Delta [27,28,29,40,41,42,43].

The primary objective of this study was to evaluate the effectiveness of COVID-19 vaccination before and after the emergence of the Omicron variant in individuals with MetS. The analysis considers vaccination schedules and hypothesizes that vaccine effectiveness—measured by protection against infection, hospitalization, and death—declined following the emergence of Omicron, with differential impacts observed in patients with and without MetS.

## 2. Materials and Methods

A retrospective cross-sectional observational study was carried out in which suspected cases of COVID-19 infection from 66 hospitals in northeastern Mexico belonging to the Mexican Social Security Institute, were included. A database with all suspected cases of COVID-19 infection reported between 5 August 2020 and 31 May 2023, was cleaned according to selection criteria, which is as follows: Records with complete information, test results for COVID-19 rapid antigen or quantitative reverse transcription polymerase chain reaction (qRT-PCR) and a previous diagnosis of diabetes mellitus, hypertension and obesity were included; those with a positive qRT-PCR test for viruses other than COVID-19 were eliminated. A total of 3194 cases were further analyzed achieving a statistical power over 95% for a confidence level of 95%. This research was approved by the Ethics and Research Committee of the Mexican Social Security Institute (Registration number R-2022-190-118).

Intentional nonprobability sampling was used for this study. All data included in this study were obtained from the clinical records and provided by the Medical Epidemiological Assistance Coordination of the Mexican Social Security Institute of the State of Nuevo Leon.

**Figure 1:**
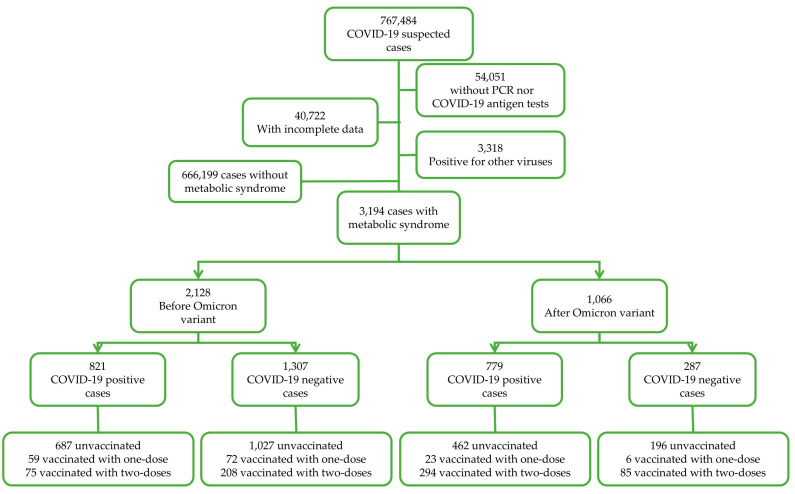
Study design.

Data subgrouping was performed for: (a) COVID-19 test results as positive and negative (according to COVID-19 rapid antigen test or qRT-PCR), (b) for the period of COVID-19 test, in which cases from 2020 to 2021 corresponded to the before Omicron (BO) period and cases from 2022 to 2023 corresponded to the after Omicron (AO) period, as described in other studies [44,45], and (c) the vaccination status (unvaccinated, one dose, and two doses). Data depuration is shown in Figure 1.

### 2.1. Study Variables

#### 2.1.1. Type of Vaccine

Type of vaccine was obtained from epidemiological records. Patients were vaccinated with the following vaccines:

Ad5-nCoV (CanSinoBIO), a one-dose regimen, recombinant, adenoviral vector vaccine with a reported effectiveness of 70% against severe/critical COVID-19 and 100% against death in real-world studies [46].

Ad26.CoV2.S (Johnson & Johnson/Janssen), a one-dose regimen recombinant vaccine designed on an adenovirus type 26 vector, which encodes the full spike protein of SARS-CoV-2 [47]. This vaccine has a reported effectiveness of 20% against COVID-19 symptoms, 43% against hospitalization and 53% against mortality in a study carried out in United Stated of America during the circulation of Alpha, Delta, and Omicron BA.1, BA.2, BA.212.1, and BA.5 variants [48].

BBIBP-CorV (Sinopharm) is a two-dose regimen, inactivated vaccine. Its effectiveness against severe disease is 84%, 73.3%, 69.4% and 66% for patients during the Alpha, Gamma, Delta and Omicron variants, respectively, in real-world studies [49].

BNT162b2 (Pfizer-BioNTech), a two-dose regimen, mRNA vaccine, with a reported effectiveness of 43% against hospitalization and 51% against death in real-world studies before Omicron [50], and 90.7% against death during Omicron circulation in 65-year-old patients and older [51].

ChAdOx1 (AstraZeneca) is a two-dose regimen adenoviral vector vaccine with a reported effectiveness of 92% for hospitalization and 91% against mortality [52].

CoronaVac (Sinovac Life Sciences) is a two-dose regimen vaccine, inactivated whole virus vaccine, created from African green monkey kidney cells (Vero cells) that were inoculated with SARS-CoV-2 (CN02 strain). The effectiveness in real-world studies has been reported to be 87.5% against hospitalization, 90.3% against ICU admission, and 86.3% against death [53].

Gam-COVID-Vac (Gamaleya’s Sputnik V) is a two-dose regimen, viral vector vaccine with a reported effectiveness of 86.8% against hospitalization, 91.9% against severe disease and 92.0% against death [54].

mRNA-1273 (Moderna) is a two-dose mRNA vaccine with a reported effectiveness of 95.8% against hospitalization or in-hospital death [55].

NVX-CoV2373 (Novavax) is a two-dose regimen, recombinant protein vaccine with a reported effectiveness in clinical trials of 90% against symptomatic disease in adults [56].

Due to low numbers, only patients vaccinated with BNT162b2 (Pfizer-BioNTech), ChAdOx1 (AstraZeneca), CoronaVac (Sinovac Life Sciences) were considered for vaccine effectiveness analysis.

#### 2.1.2. Vaccination Status

Vaccination status was obtained from epidemiological records. The subgroups were non-vaccinated, one dose with 14 days or more between vaccination and the onset of symptoms, two doses with 14 days between vaccination and the onset of symptoms.

#### 2.1.3. Vaccine Effectiveness (VE)

Three main outcomes were evaluated as dichotomic “yes” or “no” variables: (a) infection, (b) hospitalization and (c) death due to COVID-19 complications. VE was calculated with the following formula: VE  =  (1  −  OR)  ×  100% widely reported previously [27,28,57,58,59,60,61].

#### 2.1.4. Metabolic Syndrome (MetS)

Comorbidities were obtained from epidemiological records. Metabolic syndrome diagnosis criteria were established according to the Harmonized definition and American Heart Association [62,63], based on a previous diagnosis of obesity with diabetes and hypertension, at the date of COVID-19 test. Criteria for obesity was a waist circumference ≥35 inches (88 cm) in women and ≥40 inches (102 cm) in men [64,65]. Diabetes was defined as fasting glucose 100 mg/dL or more or in treatment for elevated glucose. A systolic blood pressure ≥130 mm/Hg or diastolic blood pressure ≥85 mm/Hg or drug treatment for elevated pressure was considered hypertension [66,67].

#### 2.1.5. Symptoms

Symptoms were obtained from an epidemiological database as dichotomic variables, which are presented as frequencies and percentages with estimated confidence intervals of 95%.

#### 2.1.6. Control Variables

Age, sex, tobacco smoking and antiviral use were included as control variables in the multivariate regression analysis and were obtained from epidemiological records.

#### 2.1.7. Statistical Analysis

Categoric variables such as socio-demographic data and symptoms were described as frequencies and percentages; bivariate analyses were performed using the Chi-square or Fisher’s exact test. For quantitative variables such as age, the mean and 95% confidence interval were calculated. Multivariate binary logistic regression models were integrated to identify the vaccination status as an independent predictor of COVID-19 infection, hospitalization and death as outcome variables. Different models were adjusted for age, sex, tobacco smoking and antiviral use, as shown in table footnotes. From this analysis, odds ratios (OR) and 95% confidence intervals (CI) for each outcome of interest were calculated. Data were compiled and depurated in Excel (Microsoft, Redmond, WA, USA). Data were analysed in SPSS V26 (International Business Machines Coorporation, Armonk, NY, USA). 

## 3. Results

### 3.1. Socio-Demographic Characteristics

A total of 3194 cases with MetS were collected from which 67.9% were female, with a mean age of 55 (95% CI 54.6–55.5) and mostly employed or entrepreneurs (55.9%). Regarding vaccination, patients were mostly non-vaccinated (74.3%), and vaccinated patients were mainly vaccinated with the ChAdOx1 vaccine (10.4%). Most patients recovered (90.6%) (Table 1).

### 3.2. Vaccine Effectiveness Against SARS-CoV-2 Infection in Patients with Metabolic Syndrome

Vaccine effectiveness against infection was evaluated before (BO) and after Omicron (AO), corresponding to 2020–2021 and 2022–2023, respectively. First, we evaluated the effectiveness after one dose, and we found that ChAdOx1 and CoronaVac were not effective BO and AO, whereas BNT162b2 was significantly effective only in BO patients with MetS (Table S1, unadjusted results in Table S2).

Then, an analysis was performed to evaluate the effectiveness against infection in patients with two doses of COVID-19 vaccines and this shows that BO, BNT162b2 is significantly effective whereas ChAdOx1 and CoronaVac demonstrate a non-significant protection. AO none of the vaccines protected patients with metabolic syndrome against infection (Table 2).

### 3.3. Vaccine Effectiveness Against Hospitalization in Patients with Metabolic Syndrome

Another outcome of interest was hospitalization; therefore, an analysis of the effectiveness of the COVID-19 vaccination was performed in patients with one dose and two doses of the COVID-19 vaccine. BO, ChAdOx1 was significantly effective in patients who received only one dose, a trend toward protection was observed with BNT162b2 and CoronaVac. AO, an effectiveness of 100% was observed in patients vaccinated with ChAdOx1 and CoronaVac (Table S1, unadjusted results in Table S2).

Regarding patients with two doses, BO, all analyzed vaccines significantly protected against hospitalization, whereas AO, only BNT162b2 (*p* < 0.01) and CoronaVac protected against hospitalization (Table 3).

### 3.4. Vaccine Effectiveness Against Death in Patients with Metabolic Syndrome

A similar analysis was performed for death, where BO, one dose of CoronaVac showed 100% protection against death whereas the ChAdOx1 and BNT162b2 vaccines only showed a trend toward protection against death. AO, a 100% protection against death was observed with ChAdOx1 and CoronaVac (Table S1, unadjusted results in Table S2).

Finally, an analysis of COVID-19 vaccine effectiveness against death was performed in patients with two doses and this demonstrated that BO, only BNT162b2 significantly protects patients with metabolic syndrome against death whereas ChAdOx1 and CoronaVac failed to reach significance. AO, a 100% effectiveness against death was observed with BNT162b and CoronaVac (Table 4).

### 3.5. Symptomatology in Two-Dose-Vaccinated and Non-Vaccinated Patients with Metabolic Syndrome

Before Omicron, after two doses of the ChAdOx1 vaccine, a significant increase in sore throats, running noses, and conjunctivitis was observed in the two-dose-vaccinated patients (Table S3), whereas AO, an increase in sore throat, malaise, running nose and chills was observed in two-dose-vaccinated patients (Table S3).

Regarding patients vaccinated with two doses of BNT162b2, BO, a reduction in cough, dyspnea and chest pain was observed in two-dose-vaccinated patients whereas an increased presentation of sore throat was observed in the same subgroup BO and AO (Table S4). After Omicron, the presentation of dyspnea was also reduced in two-dose-vaccinated patients (Table S4).

Finally, BO, in patients vaccinated with two doses of CoronaVac, sore throat was increased whereas, in this same subgroup, dyspnea was reduced (Table S5). AO, an increased presentation of chills was observed in two-dose-vaccinated patients (Table S5).

## 4. Discussion

A retrospective cross-sectional observational study was carried out which allows for the comparison of patients within the same period, in contact with the same SARS-CoV-2 variants given that cases took place in the same region in Mexico. The analysis carried out herein demonstrates that the effectiveness of COVID-19 vaccines has been impacted by the circulation of the SARS-CoV-2 variant Omicron in patients with MetS. Moreover, only two doses of the BNT162b2 COVID-19 vaccine reduced the presentation of dyspnea before and after Omicron.

### 4.1. Impact of Metabolic Syndrome on COVID-19 Severity

The COVID-19 pandemic has highlighted the vulnerability of individuals with MetS, particularly in the context of SARS-CoV-2 infection. A recent meta-analysis carried out in a total of 209,569 COVID-19-positive patients demonstrated that MetS is a major comorbidity, which is present in about 20% of COVID-19 patients and a 230% increased risk of short-term mortality is reported in MetS patients with COVID-19 [68]. Studies have demonstrated that MetS exacerbates the severity of COVID-19, leading to higher rates of hospitalization, intensive care unit (ICU) admissions and mortality [37,69,70]. In addition, patients with MetS are more likely to develop severe conditions such as respiratory failure and acute respiratory distress syndrome (ARDS), needing invasive clinical interventions, including mechanical ventilation [37,71].

The diseases associated with MetS have been shown to independently increase severe COVID-19 risk as stated in recent reports where it was found that males with higher levels of visceral fat were at an increased risk of hospitalization and severe disease [9,72,73].

Furthermore, an increased risk for COVID-19 infection has been observed in patients with hypertension, obesity, and diabetes with an adjusted OR of 2.54, 2.20, and 1.41, respectively [73].

Further insights confirm that obesity is a significant risk factor for severe COVID-19 outcomes since obesity exacerbates immune dysregulation and chronic inflammation, leading to complications such as acute respiratory distress syndrome (ARDS) and multi-organ failure [74], diabetes has been also related to COVID-19 severity [9,75].

In our study, we observed that unvaccinated patients with MetS exhibit an increased severity of COVID-19 symptoms, particularly those related to respiratory distress, such as dyspnea.

### 4.2. Metabolic Syndrome and Immunological Response to COVID-19

Patients with MetS experience worse outcomes from COVID-19 because of a combination of chronic inflammation and endothelial dysfunction, key mechanisms in driving severe disease [76]. Obesity exacerbates this inflammatory response, as demonstrated by Johnson et al. (2023) findings where they reported that diet-induced obesity was associated with the severity of respiratory symptoms in a COVID-19 mouse model, further supporting the observed association between MetS and exacerbated COVID-19 symptoms in our study [77]. The disruption of B-cell and T-cell functions in this inflammatory environment reduces the production of neutralizing antibodies and weakens cellular immunity [23,78,79]. Additionally, diabetes contributes to a delayed and weakened T-cell response [78,80]. These factors combined result in a faster decline in antibody titers in MetS patients, regardless of vaccine type [17].

Impaired immune regulation in MetS patients affects their response to infection. Chronic inflammation in MetS can lead to an overactive but inefficient immune response during SARS-CoV-2 infection, resulting in severe symptoms [81,82]. In contrast, healthier individuals with stronger and longer-lasting immune responses both to infection and vaccination, with lower rates of breakthrough infections and better long-term immunity [83,84]. This highlights the need for tailored vaccination strategies, including more frequent booster doses, to sustain protective immunity in this vulnerable population [85,86].

### 4.3. COVID-19 Vaccination Effectiveness in Comorbid Patients

Contrasting results have been found regarding the durability of vaccine-induced immunity in comorbid patients. Das et al. (2023) found that regardless of the vaccine type, all of them possess the potential to rapidly induce protective immunity against SARS-CoV-2 and demonstrated that patients with chronic diseases such as diabetes and kidney disease experienced a faster decline in antibody levels compared to healthy individuals, potentially reducing long-term vaccine effectiveness [17]. On the other hand, a study by Riyyan et al. (2022), which included various vaccines suggested that the presence of comorbidities does not impact vaccine safety nor increase the incidence of adverse reactions [87], while Thirión-Romero et al. (2023) demonstrated that in patients vaccinated with ChAdOx1 nCoV-19, Gam-COVID-Vac or BNT162b2, the effectiveness in preventing hospitalization with any complete schedule was 73% independent of comorbidity [87,88].

Regarding COVID-19 effectiveness in patients with MetS, there is limited information. There is one study carried out in China in a total of 316 individuals, from which 111 were diagnosed with MetS, all recruited during the Omicron period, and they demonstrate an intensive care unit admission rate of 66.7% for unvaccinated patients and 19.2% for those with two doses of BBIBP-CorV, CoronaVac, or other [89]. Another study investigated the safety and immunogenicity of inactivated COVID-19 in 157 adult patients with MetS and they demonstrated a poorer response in MetS patients compared to healthy controls [90].

Our results confirm that vaccines remain effective in preventing severe outcomes in patients with MetS, although they demonstrated a higher likelihood of infections. We observed that despite initial immunity from mRNA vaccines, patients with MetS and associated conditions might have experienced faster waning immunity and possibly require more frequent booster doses to maintain protection. This was also reported by other studies [17,91].

### 4.4. Omicron and Vaccine Effectiveness

The emergence of the Omicron variant significantly reduced the effectiveness of COVID-19 vaccines, particularly in individuals with underlying conditions. Vaccines that initially showed strong protection against earlier variants, experienced a notable decline in preventing infection with Omicron, with breakthrough infections becoming more common [27,29]. It was shown that the Omicron variant has certain abilities to escape naturally acquired and vaccine-induced immunity and that, compared to the Delta variant, the Omicron variant needs 10-fold increased antibody titer to be neutralized, after vaccination with ChAdOx1 or BNT162b2 [92]. Reasons for the reduction of vaccine effectiveness against Omicron have been explored before where the mutations and deletions of the Spike protein, against which antibodies and vaccines are directed, could be the reason why Omicron can evade humoral immune responses [93]. Several studies have reported the neutralizing capacity of vaccination against the Omicron variant and a reduction in vaccine effectiveness of 8–127 times was shown [93]. Furthermore, Omicron subvariants can escape the immune response generated by a previous infection with a different Omicron subvariant [94].

Research by Gan et al. (2024) and Kanokudom et al. (2024) indicated that booster doses significantly increased neutralizing antibody levels, improving protection against Omicron. However, the durability of this protection remains a concern, particularly in populations with MetS, where immune responses may be inherently weaker or less durable [95,96].

In our study, we observed a significant reduction in vaccine effectiveness against infection post-Omicron in MetS patients. This aligns with the findings of Spiteri et al. (2024) and Lai et al., (2024) who reported diminished vaccine efficacy during the Omicron wave in patients with different comorbidities [97,98].

However, our data also revealed that certain vaccines, such as BNT162b2, continued to provide substantial protection against severe outcomes, including hospitalization and death, despite the reduced effectiveness against infection. This suggests that while Omicron has impacted vaccines’ effectiveness against infection, they remain crucial in preventing severe disease, especially in high-risk groups kike those with MetS. 

### 4.5. Metabolic Syndrome and Post-COVID-19 Complications

MetS has been linked to a higher risk of developing long COVID and associated complications. Studies have shown that individuals with obesity exhibit prolonged and more severe post-COVID-19 symptoms, including respiratory and cardiovascular issues when compared to non-obese individuals [99]. Insulin resistance and altered body composition, often seen in MetS patients, further exacerbate the long-term impact of COVID-19, with symptoms and higher rates of complications such as fatigue, breathlessness, and sleep disorders [100]. These findings underscore the importance of early intervention and tailored treatment strategies for MetS patients to mitigate the risk of long COVID and the associated long-term health complications.

### 4.6. Limitations

There were no data on antibody levels for vaccinated patients; therefore, the waning of the immune response could have influenced vaccine effectiveness rates. This study collected data from only one out of the 32 states of Mexico, this compromises the generalizability of the study. Previous COVID-19 infections could have impacted patient response to COVID-19 disease. Unfortunately, this study did not count any information regarding Omicron subvariants; however, other studies have shown that COVID-19 vaccine effectiveness is reduced in Omicron-infected patients compared to Delta-infected patients, agreeing with our results.

## 5. Conclusions

We demonstrated that before Omicron, vaccine effectiveness against infection was poor with only one vaccine (BNT162b2) demonstrating to be effective with one dose or two doses. This effectiveness was lost or decreased against infection after Omicron. Regarding hospitalization and death, vaccines were still effective. Our results allow us to propose a tailored vaccination scheme for patients with MetS given the varying effectiveness rates observed in our study depending on the vaccine.

## Figures and Tables

**Table 1:** Socio-demographic and other characteristics.

		*COVID-19*		
Characteristics	Total	Yes (*n* = 1600)	No (*n* = 1594)	Chi-Square *p*-Value
*Sex*				
Female	2169 (67.9)	1048 (65.5)	1121 (70.3)	0.003
Male	1025 (32.1)	552 (34.5)	473 (29.7)	
*Age*				
≤49	1037 (32.5)	522 (32.6)	515 (32.3)	0.763
50–59	1090 (34.1)	553 (34.6)	537 (33.7)	
>59	1067 (33.4)	525 (32.8)	542 (34.0)	
*Occupation ^a^*				
Employed/Entrepreneur	1785 (55.9)	914 (57.2)	871 (54.6)	0.036
House maker	982 (30.8)	468 (29.3)	514 (32.2)	
Retired/Unemployed	420 (13.2)	216 (13.5)	204 (12.8)	
Student	5 (0.2)	0 (0.0)	5 (0.3)	
*Vaccination*				
Non-vaccinated	2372 (74.3)	1149 (71.8)	1223 (76.7)	0.004
One dose	160 (5.0)	82 (5.1)	78 (4.9)	
Two doses	662 (20.7)	369 (23.1)	293 (18.4)	
*Vaccine*				
ChAdOx1	333 (40.5)	199 (44.1)	134 (8.4)	<0.001
BNT162b2	310 (37.7)	148 (32.9)	162 (10.2)	
CoronaVac	152 (18.5)	91 (20.1)	61 (3.8)	
Other	27 (0.9)	13 (0.8)	14 (0.9)	
*Outcome*				
Recovery	2869 (90.6)	1358 (86.0)	1511 (95.2)	<0.001
Death	298 (9.4)	221 (14.0)	77 (4.8)	

^a^ Occupation was not disclosed by two vaccinated patients.

**Table 2:** Two doses of COVID-19 vaccine effectiveness against infection before and after Omicron.

***Period***	***Vaccine Effectiveness (1-OR ^a^) (%)***		
	ChAdOx1	BNT162b2	CoronaVac
BO	12.9%	72% ***	18.2%
AO	−20.3%	−75.1% *	−70.7%

* *p* < 0.05, *** *p* < 0.001, OR—Odd Ratio, ^a^—OR adjusted for sex, age, tobacco smoking and antiviral use. Exact *p*-values are described in Supplementary Table S1.

**Table 3:** Two doses of COVID-19 vaccine effectiveness against hospitalization before and after Omicron.

***Period***	***Vaccine Effectiveness (1-OR ^a^) (%)***		
	ChAdOx1	BNT162b2	CoronaVac
BO	69.5% ***	71.9% ***	85.1 ***
AO	34.4%	72.2% **	100%

** *p* < 0.01, *** *p* < 0.001, OR—Odd Ratio, ^a^—OR adjusted for sex, age, tobacco smoking and antiviral use. Exact *p*-values are described in Supplementary Table S1.

**Table 4:** Two doses of COVID-19 vaccine effectiveness against death before and after Omicron.

***Period***	***Vaccine Effectiveness (1-OR ^a^) (%)***		
	ChAdOx1	BNT162b2	CoronaVac
BO	37.7%	80.3% ***	51.7%
AO	19.1	100%	100%

*** *p* < 0.001, OR—Odd Ratio, ^a^—OR adjusted for sex, age, tobacco smoking and antiviral use. Exact *p*-values are described in Supplementary Table S1.

## Data Availability

Data are available upon reasonable request to the corresponding author due to privacy or ethical restrictions.

## Supplementary Materials

The following supporting information can be downloaded at: https://www.mdpi.com/article/10.3390/vaccines13030244/s1, Table S1 COVID-19 vaccines effectiveness in patients with metabolic syndrome before and after Omicron. Table S2 Unadjusted COVID-19 vaccines effectiveness in patients with metabolic syndrome before and after Omicron. Table S3 Symptom presentation in unvaccinated patients and patients vaccinated with two doses of ChAdOx1 vaccine. Table S4 Symptom presentation in unvaccinated patients and patients vaccinated with two doses of BNT162b2. Table S5 Symptom presentation in unvaccinated patients and patients vaccinated with two doses of CoronaVac.
