# Supplementary material for: Assessment of COVID-19 Vaccine Effectiveness Against SARS-CoV-2 Infection, Hospitalization and Death in Mexican Patients with Metabolic Syndrome from Northeast Mexico: A Multicenter Study
Source: Vaccines (Basel). 2025 Feb 27;13(3):244. doi: 10.3390/vaccines13030244 (PMC11945729; doi:10.3390/vaccines13030244)
Supplement: Supplementary file 1 [file vaccines-13-00244-s001.zip › Table S1.pdf]

Supplementary Table S1. COVID-19 vaccines effectiveness in patients with metabolic syndrome before and after Omicron.

Before Omicron (2020-2021)

		COVID-19 infection				Hospitalization				Death			
	**Total**	**Yes**	**No**	**Effectiveness (95%CI) (Adjusted 1 – OR)**	***p*-value**	**Yes**	**No**	**Effectiveness (95%CI) (Adjusted 1 – OR)**	***p*-value**	**Yes**	**No**	**Effectiveness (95%CI) (Adjusted 1 – OR)**	***p*-value**
**BNT162b2 (Pfizer)**													
No vaccine	1,714 (91.4)	687 (95.7)	1,027 (88.7)	Ref.		583 (95.4)	1,131 (89.4)	Ref.		253 (97.7)	1,450 (90.3)	Ref.	
1st dose 0-13 days	4 (0.2)	3 (0.4)	1 (0.1)	-358.6% (-4335.9%,52.6%)	0.188	2 (0.3)	2 (0.2)	-118.3% (-1525.4%,70.7%)	0.446	0 (0.0)	4 (0.2)	100%	0.999
1st dose ≥14 days	25 (1.3)	5 (0.7)	20 (1.7)	63.4% (1.4%,86.4%)	0.047	6 (1.0)	19 (1.5)	18.7% (-112.8%,68.9%)	0.673	1 (0.4)	24 (1.5)	67.9% (-144.1%,95.8%)	0.272
2nd dose 0-13 days	9 (0.5)	3 (0.4)	6 (0.5)	27.7% (-191.8%,82.1%)	0.649	1 (0.2)	8 (0.6)	77.5% (-86.2%,97.3%)	0.167	0 (0.0)	9 (0.6)	100%	0.999
2nd dose ≥14 days	124 (6.6)	20 (2.8)	104 (9.0)	72% (54.2%,82.8%)	<0.001	19 (3.1)	105 (8.3)	71.9% (52.9%,83.3%)	<0.001	5 (1.9)	119 (7.4)	80.3% (50.7%,92.1%)	<0.001
**ChAdOx1 (Astra-Zeneca)**													
No vaccine	1,714 (91.7)	687 (91.5)	1,027 (91.9)	Ref.		583 (96.4)	1,131 (89.5)	Ref.				Ref.	
1st dose 0-13 days	14 (0.7)	10 (1.3)	4 (0.4)	-332.4% (-1292.5%,-34.3%)	0.014	2 (0.3)	12 (0.9)	43% (-160.3%,87.5%)	0.469	253 (95.1)	1,450 (91.2)	8.4% (-618.5%,88.3%)	0.933
1st dose ≥14 days	54 (2.9)	24 (3.2)	30 (2.7)	-31.5% (-128.6%,24.3%)	0.331	6 (1.0)	48 (3.8)	70.2% (27.9%,87.6%)	0.007	1 (0.4)	13 (0.8)	52.7% (-56.5%,85.7%)	0.22
2nd dose 0-13 days	16 (0.9)	4 (0.5)	12 (1.1)	50.8% (-53.7%,84.3%)	0.222	2 (0.3)	14 (1.1)	100%	0.042	3 (1.1)	50 (3.1)	67.7% (-152%,95.8%)	0.281
2nd dose ≥14 days	71 (3.8)	26 (3.5)	45 (4.0)	12.9% (-43.2%,47%)	0.586	12 (2.0)	59 (4.7)	69.5% (41.8%,84%)	<0.001	1 (0.4)	15 (0.9)	37.7% (-34.1%,71%)	0.227
**CoronaVac (Sinovac)**													
No vaccine	1,174 (95.4)	687 (95.2)	1,027 (95.6)	Ref.		583 (98.8)	1,131 (93.8)	Ref.		253 (98.4)	1,450 (94.9)	Ref.	
1st dose 0-13 days	3 (0.2)	1 (0.1)	2 (0.2)	24.2% (-742.9%,93.2%)	0.822	0 (0.0)	3 (0.2)	100%	0.999	0 (0.0)	3 (0.2)	100%	0.999
1st dose ≥14 days	19 (1.1)	12 (1.7)	7 (0.7)	-179.1% (-615.4%,-8.9%)	0.033	3 (0.5)	16 (1.3)	49.9% (-75.2%,85.7%)	0.279	0 (0.0)	19 (1.2)	100%	0.998
2nd dose 0-13 days	6 (0.3)	3 (0.4)	3 (0.3)	-47.1% (-636.7%,70.6%)	0.639	0 (0.0)	6 (0.5)	100%	0.999	0 (0.0)	6 (0.4)	100%	0.999
2nd dose ≥14 days	54 (3.0)	19 (2.6)	35 (3.3)	18.2% (-44.5%,53.7%)	0.488	4 (0.7)	50 (4.1)	85.1% (57.9%,94.7%)	<0.001	4 (1.6)	50 (3.3)	51.7% (-37.6%,83%)	0.173

After Omicron (2022-2023)

		COVID-19 infection				Hospitalization				Death			
	**Total**	**Yes**	**No**	**Effectiveness (95%CI) (Adjusted 1 – OR)**	***p*-value**	**Yes**	**No**	**Effectiveness (95%CI) (Adjusted 1 – OR)**	***p*-value**	**Yes**	**No**	**Effectiveness (95%CI) (Adjusted 1 – OR)**	***p*-value**
**BNT162b2 (Pfizer)**													
No vaccine	658 (81.6)	462 (79.8)	196 (86.3)	Ref.		88 (92.6)	570 (80.2)	Ref.		17 (94.4)	633 (81.5)	Ref.	
1st dose ≥14 days	10 (1.2)	8 (1.4)	2 (0.9)	-23.6% (-592.1%,77.9%)	0.810	1 (1.1)	9 (1.3)	17.2% (-770.6%,92.1%)	0.875	1 (5.6)	8 (1.0)	-1236% (-19148.8%,7.3%)	0.057
2nd dose ≥14 days	138 (17.1)	109 (18.8)	29 (12.8)	-75.1% (-173.7%,-10.8%)	0.016	6 (6.3)	132 (18.6)	72.2% (34%,88.3%)	0.004	0 (0.0)	136 (17.5)	100%	0.996
**ChAdOx1 (Astra-Zeneca)**													
No vaccine	658 (78.7)	462 (77.4)	196 (82.0)	Ref.		88 (86.3)	570 (77.7)	Ref.		17 (85.0)	633 (78.6)	Ref.	
1st dose ≥14 days	15 (1.8)	12 (2.0)	3 (1.3)	-48.3% (-481.9%,62.2%)	0.572	0 (0.0)	15 (2.0)	100%	0.998	0 (0.0)	15 (1.9)	100%	0.999
2nd dose ≥14 days	163 (19.5)	123 (20.6)	40 (16.7)	-20.3% (-81.5%,20.2%)	0.378	14 (13.7)	149 (20.3)	34.4% (-22.3%,64.8%)	0.185	3 (15.0)	157 (19.5)	19.1% (-187.8%,77.3%)	0.743
**CoronaVac (Sinovac)**													

No vaccine	658 (90.4)	462 (89.2)	196 (93.3)	Ref.		88 (100.0)	570 (89.1)	Ref.		17 (100.0)	633 (90.0)	Ref.	
1st dose ≥14 days	2 (0.3)	2 (0.4)	0 (0.0)	0%	0.999	0 (0.0)	2 (0.3)	100%	0.999	0 (0.0)	2 (0.3)	100%	1
2nd dose ≥14 days	68 (9.3)	54 (10.4)	14 (6.7)	-70.7% (-218.8%,8.6%)	0.093	0 (0.0)	68 (10.6)	100%	0.997	0 (0.0)	68 (9.7)	100%	0.997

OR – Odd ratios, OR adjusted for sex, age and tobacco smoking and antiviral use.
