# Supplementary material for: Assessment of COVID-19 Vaccine Effectiveness Against SARS-CoV-2 Infection, Hospitalization and Death in Mexican Patients with Metabolic Syndrome from Northeast Mexico: A Multicenter Study
Source: Vaccines (Basel). 2025 Feb 27;13(3):244. doi: 10.3390/vaccines13030244 (PMC11945729; doi:10.3390/vaccines13030244)
Supplement: Supplementary file 1 [file vaccines-13-00244-s001.zip › Table S2.pdf]

# Supplementary Table S2. Unadjusted COVID-19 vaccines effectiveness in patients with metabolic syndrome before and after Omicron.

| | | | | | | | | | | | | | |
|---|---|---|---|---|---|---|---|---|---|---|---|---|---|
| **Before Omicron (2020-2021)** | | | | | | | | | | | | | |
| | | **COVID-19 infection** | | | | **Hospitalization** | | | | **Death** | | | |
| | **Total** | **Yes** | **No** | **Effectiveness (95%CI) (Adjusted 1 – OR)** | ***p*-value** | **Yes** | **No** | **Effectiveness (95%CI) (Adjusted 1 – OR)** | ***p*-value** | **Yes** | **No** | **Effectiveness (95%CI) (Adjusted 1 – OR)** | ***p*-value** |
| **BNT162b2 (Pfizer)** | | | | | | | | | | | | | |
| No vaccine | 1,714 (91.4) | 687 (95.7) | 1,027 (88.7) | Ref. | | 583 (95.4) | 1,131 (89.4) | Ref. | | 253 (97.7) | 1,450 (90.3) | Ref. | |
| 1st dose 0-13 days | 4 (0.2) | 3 (0.4) | 1 (0.1) | -348.5% (-4220.3%,53.4%) | 0.194 | 2 (0.3) | 2 (0.2) | -94% (-1280.7%,72.7%) | 0.508 | 0 (0.0) | 4 (0.2) | 100% | 0.999 |
| 1st dose ≥14 days | 25 (1.3) | 5 (0.7) | 20 (1.7) | 62.6% (-0.1%,86%) | 0.05 | 6 (1.0) | 19 (1.5) | 38.7% (-54.2%,75.7%) | 0.298 | 1 (0.4) | 24 (1.5) | 76.1% (-77.3%,96.8%) | 0.016 |
| 2nd dose 0-13 days | 9 (0.5) | 3 (0.4) | 6 (0.5) | 25.3% (-199.9%,81.4%) | 0.681 | 1 (0.2) | 8 (0.6) | 75.8% (-94.3%,97%) | 0.182 | 0 (0.0) | 9 (0.6) | 100% | 0.999 |
| 2nd dose ≥14 days | 124 (6.6) | 20 (2.8) | 104 (9.0) | 71.3% (53.2%,82.4%) | <0.001 | 19 (3.1) | 105 (8.3) | 64.9% (42.2%,78.7%) | <0.001 | 5 (1.9 | 119 (7.4) | 75.9% (40.5%,90.3%) | 0.002 |
| **ChAdOx1 (AstraZeneca)** | | | | | | | | | | | | | |
| No vaccine | 1,714 (91.7) | 687 (91.5) | 1,027 (91.9) | Ref. | | 583 (96.4) | 1,131 (89.5) | Ref. | | | | Ref. | |
| 1st dose 0-13 days | 14 (0.7) | 10 (1.3) | 4 (0.4) | -273.7% (-1096.4%,-16.7%) | 0.026 | 2 (0.3) | 12 (0.9) | 67.7% (-44.9%,92.8%) | 0.14 | 253 (95.1) | 1,450 (91.2) | 55.9% (-238.5%,94.3%) | 0.431 |
| 1st dose ≥14 days | 54 (2.9) | 24 (3.2) | 30 (2.7) | -19.6% (-106.3%,30.7%) | 0.52 | 6 (1.0) | 48 (3.8) | 75.8% (43%,89.7%) | 0.001 | 1 (0.4) | 13 (0.8) | 65.6% (-11.1%,89.4%) | 0.074 |
| 2nd dose 0-13 days | 16 (0.9) | 4 (0.5) | 12 (1.1) | 50.2% (-55.1%,84%) | 0.229 | 2 (0.3) | 14 (1.1) | 100% | 0.09 | 3 (1.1) | 50 (3.1) | 61.8% (-190.5%,95%) | 0.353 |
| 2nd dose ≥14 days | 71 (3.8) | 26 (3.5) | 45 (4.0) | 13.6% (-41.3%,47.2%) | 0.56 | 12 (2.0) | 59 (4.7) | 60.5% (26%,79%) | 0.004 | 1 (0.4) | 15 (0.9) | 26% (-56.3%,65%) | 0.429 |
| **CoronaVac (Sinovac)** | | | | | | | | | | | | | |
| No vaccine | 1,174 (95.4) | 687 (95.2) | 1,027 (95.6) | Ref. | | 583 (98.8) | 1,131 (93.8) | Ref. | | 253 (98.4) | 1,450 (94.9) | Ref. | |
| 1st dose 0-13 days | 3 (0.2) | 1 (0.1) | 2 (0.2) | 25.3% (-725.9%,93.2%) | 0.812 | 0 (0.0) | 3 (0.2) | 100% | 0.999 | 0 (0.0) | 3 (0.2) | 100% | 0.999 |
| 1st dose ≥14 days | 19 (1.1) | 12 (1.7) | 7 (0.7) | -156.3% (-554.2%,-0.4%) | 0.049 | 3 (0.5) | 16 (1.3) | 63.6% (-25.3%,89.4%) | 0.109 | 0 (0.0) | 19 (1.2) | 100% | 0.998 |
| 2nd dose 0-13 days | 6 (0.3) | 3 (0.4) | 3 (0.3) | -49.5% (-642.8%,69.9%) | 0.623 | 0 (0.0) | 6 (0.5) | 100% | 0.999 | 0 (0.0) | 6 (0.4) | 100% | 0.999 |
| 2nd dose ≥14 days | 54 (3.0) | 19 (2.6) | 35 (3.3) | 18.8% (-43%,54%) | 0.47 | 4 (0.7) | 50 (4.1) | 84.5% (56.8%,94.4%) | <0.001 | 4 (1.6) | 50 (3.3) | 54.2% (-28.1%,83.6%) | 0.137 |
| **After Omicron (2022-2023)** | | | | | | | | | | | | | |
| | | **COVID-19 infection** | | | | **Hospitalization** | | | | **Death** | | | |
| | **Total** | **Yes** | **No** | **Effectiveness (95%CI) (Adjusted 1 – OR)** | ***p*-value** | **Yes** | **No** | **Effectiveness (95%CI) (Adjusted 1 – OR)** | ***p*-value** | **Yes** | **No** | **Effectiveness (95%CI) (Adjusted 1 – OR)** | ***p*-value** |
| **BNT162b2 (Pfizer)** | | | | | | | | | | | | | |
| No vaccine | 658 (81.6) | 462 (79.8) | 196 (86.3) | Ref. | | 88 (92.6) | 570 (80.2) | Ref. | | 17 (94.4) | 633 (81.5) | Ref. | |
| 1st dose ≥14 days | 10 (1.2) | 8 (1.4) | 2 (0.9) | -69.7% (-706.3%,64.3%) | 0.506 | 1 (1.1) | 9 (1.3) | 28% (-475%,91%) | 0.756 | 1 (5.6) | 8 (1.0) | -365.4% (-3832.1%,44.9%) | 0.158 |
| 2nd dose ≥14 days | 138 (17.1) | 109 (18.8) | 29 (12.8) | -59.5% (-148.2%,-2.5%) | 0.039 | 6 (6.3) | 132 (18.6) | 70.6% (31.2%,87.4%) | 0.005 | 0 (0.0) | 136 (17.5) | 100% | 0.996 |
| **ChAdOx1 (AstraZeneca)** | | | | | | | | | | | | | |
| No vaccine | 658 (78.7) | 462 (77.4) | 196 (82.0) | Ref. | | 88 (86.3) | 570 (77.7) | Ref. | | 17 (85.0) | 633 (78.6) | Ref. | |
| 1st dose ≥14 days | 15 (1.8) | 12 (2.0) | 3 (1.3) | -69.7% (-508%,52.6%) | 0.417 | 0 (0.0) | 15 (2.0) | 100% | 0.999 | 0 (0.0) | 15 (1.9) | 100% | 0.999 |
| 2nd dose ≥14 days | 163 (19.5) | 123 (20.6) | 40 (16.7) | -30.5% (-93.4%,12%) | 0.186 | 14 (13.7) | 149 (20.3) | 39.1% (-10%,66.3%) | 0.100 | 3 (15.0) | 157 (19.5) | 28.8% (-145.8%,79.4%) | 0.591 |
| **CoronaVac (Sinovac)** | | | | | | | | | | | | | |
| No vaccine | 658 (90.4) | 462 (89.2) | 196 (93.3) | Ref. | | 88 (100.0) | 570 (89.1) | Ref. | | 17 (100.0) | 633 (90.0) | Ref. | |
| 1st dose ≥14 days | 2 (0.3) | 2 (0.4) | 0 (0.0) | 0% | 0.999 | 0 (0.0) | 2 (0.3) | 100% | 0.999 | 0 (0.0) | 2 (0.3) | 100% | 1.000 |
| 2nd dose ≥14 days | 68 (9.3) | 54 (10.4) | 14 (6.7) | -63.6% (-201.5%,11.2%) | 0.114 | 0 (0.0) | 68 (10.6) | 100% | 0.997 | 0 (0.0) | 68 (9.7) | 100% | 0.997 |
