# Supplementary material for: Assessment of COVID-19 Vaccine Effectiveness Against SARS-CoV-2 Infection, Hospitalization and Death in Mexican Patients with Metabolic Syndrome from Northeast Mexico: A Multicenter Study
Source: Vaccines (Basel). 2025 Feb 27;13(3):244. doi: 10.3390/vaccines13030244 (PMC11945729; doi:10.3390/vaccines13030244)
Supplement: Supplementary file 1 [file vaccines-13-00244-s001.zip › Table S3.pdf]

Table S3. Symptom presentation in unvaccinated patients and patients vaccinated with two doses of ChAdOx1 vaccine.

	ChAdOx (BO, n = 1,785)					ChAdOx (AO, n = 821)				
**Symptoms**	**Unvaccinated (n = 1,714)**		**Two doses (>14 days) (n = 71)**			**Unvaccinated (n = 658)**		**Two doses (>14 days) (n = 163)**		
	n		n	% (95% CI)	**chi-square p-value**	n	% (95% CI)	n	% (95% CI)	**chi-square p-value**
Cough	1,189	69.4 (67.1, 71.5)	55	77.5 (66.5, 85.6)	0.146	519	78.9 (75.6, 81.8)	136	83.4 (77, 88.4)	0.194
Headache	1,156	67.4 (65.2, 69.6)	55	77.5 (66.5, 85.6)	0.077	489	74.3 (70.8, 77.5)	114	69.9 (62.5, 76.5)	0.257
Muscle pain	905	52.8 (50.4, 55.2)	39	54.9 (43.4, 66)	0.725	328	49.8 (46, 53.7)	87	53.4 (45.7, 60.9)	0.420
Fever	822	48 (45.6, 50.3)	37	52.1 (40.7, 63.3)	0.492	349	53.0 (49.2, 56.8)	94	57.7 (50, 65)	0.288
Joint Pain	792	46.2 (43.9, 48.6)	31	43.7 (32.7, 55.2)	0.673	267	40.6 (36.9, 44.4)	63	38.7 (31.5, 46.3)	0.653
Sore throat	749	43.7 (41.4, 46.1)	41	57.7 (46.2, 68.5)	0.020	353	53.6 (49.8, 57.4)	111	68.1 (60.6, 74.8)	0.001
Malaise	650	37.9 (35.7, 40.2)	30	42.3 (31.5, 53.8)	0.462	184	28.0 (24.7, 31.5)	62	38 (30.9, 45.7)	0.012
Running nose	586	34.2 (32, 36.5)	33	46.5 (35.4, 58)	0.033	327	36.0 (32.4, 39.8)	95	58.3 (50.6, 65.6)	0.050
Dyspnea	554	32.3 (30.1, 34.6)	17	23.9 (15.5, 35)	0.138	93	14.1 (11.7, 17)	20	12.3 (8.1, 18.2)	0.536
Chills	544	31.7 (29.6, 34)	26	36.6 (26.4, 48.2)	0.387	208	31.6 (28.2, 35.3)	73	44.8 (37.4, 52.5)	0.002
Chest pain	359	20.9 (19.1, 22.9)	15	21.1 (13.2, 32)	0.971	94	14.3 (11.8, 17.2)	17	10.4 (6.6, 16.1)	0.197
Anosmia	221	12.9 (11.4, 14.6)	11	15.5 (8.9, 25.7)	0.523	25	3.8 (2.6, 5.5)	7	4.3 (2.1, 8.6)	0.770
Dysgeusia	204	11.9 (10.5, 13.5)	10	14.1 (7.8, 24)	0.579	33	5.0 (3.6, 7)	7	4.3 (2.1, 8.6)	0.702
Diarrea	202	11.8 (10.3, 13.4)	7	9.9 (4.9, 19)	0.621	38	5.8 (4.2, 7.8)	9	5.5 (2.9, 10.2)	0.901
Abdominal pain	196	11.4 (10, 13)	4	5.6 (2.2, 13.6)	0.129	66	10.0 (8, 12.6)	13	8.0 (4.7, 13.2)	0.426
Conjunctivitis	47	2.7 (2.1, 3.6)	6	8.5 (3.9, 17.2)	0.017[a]	25	3.8 (2.6, 5.5)	3	1.8 (0.6, 5.3)	0.217
Prostration	34	2 (1.4, 2.8)	2	2.8 (0.8, 9.7)	0.625[a]	13	2.0 (1.2, 3.4)	6	3.7 (1.7, 7.8)	0.239[a]
Other	29	1.7 (1.2, 2.4)	0	0 (0, 5.1)	0.627[a]	2	0.3 (0.1, 1.1)	2	1.2 (0.3, 4.4)	0.178[a]
Cyanosis	17	1.0 (0.6, 1.6)	2	2.8 (0.8, 9.7)	0.173[a]	5	0.8 (0.3, 1.8)	0	0 (0, 2.3)	0.589[a]
Polypnea	17	1.0 (0.6, 1.6)	2	2.8 (0.8, 9.7)	0.173[a]	5	0.8 (0.3, 1.8)	0	0 (0, 2.3)	0.589[a]
Coriza	12	0.7 (0.4, 1.2)	0	0 (0, 5.1)	1.000[a]	3	0.5 (0.2, 1.3)	2	1.2 (0.3, 4.4)	0.259[a]

[a] Fisher exact test *p*-value.
