# Supplementary material for: Assessment of COVID-19 Vaccine Effectiveness Against SARS-CoV-2 Infection, Hospitalization and Death in Mexican Patients with Metabolic Syndrome from Northeast Mexico: A Multicenter Study
Source: Vaccines (Basel). 2025 Feb 27;13(3):244. doi: 10.3390/vaccines13030244 (PMC11945729; doi:10.3390/vaccines13030244)
Supplement: Supplementary file 1 [file vaccines-13-00244-s001.zip › Table S4.pdf]

## Table S4. Symptom presentation in unvaccinated patients and patients vaccinated with two doses of BNT162b2.

| Symptoms | BNT162b2 (BO, n = 1,838) | | | | | BNT162b2 (AO, n = 796) | | | | |
|---|---|---|---|---|---|---|---|---|---|---|
| | Unvaccinated (n = 1,714) | | Two doses (>14 days) (n = 124) | | chi-square p-value | Unvaccinated (n = 658) | | Two doses (>14 days) (n = 138) | | chi-square p-value |
| | n | % (95% CI) | n | % (95% CI) | | n | % (95% CI) | n | % (95% CI) | |
| Cough | 1,189 | 69.4 (67.1, 71.5) | 75 | 60.5 (51.7, 68.6) | 0.039 | 519 | 78.9 (75.6, 81.8) | 103 | 74.6 (66.8, 81.2) | 0.273 |
| Head ache | 1,156 | 67.4 (65.2, 69.6) | 88 | 71.0 (62.4, 78.2) | 0.418 | 489 | 74.3 (70.8, 77.5) | 99 | 71.7 (63.7, 78.6) | 0.531 |
| Muscle pain | 905 | 52.8 (50.4, 55.2) | 64 | 51.6 (42.9, 60.2) | 0.798 | 328 | 49.8 (46, 53.7) | 63 | 45.7 (37.6, 54) | 0.370 |
| Fever | 822 | 48 (45.6, 50.3) | 54 | 43.5 (35.1, 52.3) | 0.342 | 349 | 53.0 (49.2, 56.8) | 68 | 49.3 (41.1, 57.5) | 0.421 |
| Joint Pain | 792 | 46.2 (43.9, 48.6) | 53 | 42.7 (34.4, 51.5) | 0.455 | 267 | 40.6 (36.9, 44.4) | 46 | 33.3 (26, 41.6) | 0.113 |
| Sore throat | 749 | 43.7 (41.4, 46.1) | 66 | 53.2 (44.5, 61.8) | 0.039 | 353 | 53.6 (49.8, 57.4) | 89 | 64.5 (56.2, 72) | 0.020 |
| Malaise | 650 | 37.9 (35.7, 40.2) | 46 | 37.1 (29.1, 45.9) | 0.855 | 184 | 28.0 (24.7, 31.5) | 43 | 31.2 (24, 39.3) | 0.450 |
| Running nose | 586 | 34.2 (32, 36.5) | 51 | 41.1 (32.9, 49.9) | 0.117 | 327 | 49.7 (45.9, 53.5) | 74 | 53.6 (45.3, 61.7) | 0.402 |
| Dyspnea | 554 | 32.3 (30.1, 34.6) | 22 | 17.7 (12, 25.4) | 0.001 | 93 | 14.1 (11.7, 17) | 9 | 6.5 (3.5, 11.9) | 0.015 |
| Chills | 544 | 31.7 (29.6, 34) | 43 | 34.7 (26.9, 43.4) | 0.498 | 208 | 31.6 (28.2, 35.3) | 35 | 25.4 (18.8, 33.2) | 0.147 |
| Chest pain | 359 | 20.9 (19.1, 22.9) | 15 | 12.1 (7.5, 19) | 0.018 | 94 | 14.3 (11.8, 17.2) | 13 | 9.4 (5.6, 15.5) | 0.128 |
| Anosmia | 221 | 12.9 (11.4, 14.6) | 11 | 8.9 (5, 15.2) | 0.193 | 25 | 3.8 (2.6, 5.5) | 3 | 2.2 (0.7, 6.2) | 0.452 [a] |
| Dysgeusia | 204 | 11.9 (10.5, 13.5) | 9 | 7.3 (3.9, 13.2) | 0.119 | 33 | 5.0 (3.6, 7) | 1 | 0.7 (0.1, 4) | 0.023 |
| Diarrea | 202 | 11.8 (10.3, 13.4) | 14 | 11.3 (6.8, 18.1) | 0.869 | 38 | 5.8 (4.2, 7.8) | 8 | 5.8 (3, 11) | 0.992 |
| Abdominal pain | 196 | 11.4 (10, 13) | 18 | 14.5 (9.4, 21.8) | 0.302 | 66 | 10 (8, 12.6) | 7 | 5.1 (2.5, 10.1) | 0.067 |
| Conjunctivitis | 47 | 2.7 (2.1, 3.6) | 2 | 1.6 (0.4, 5.7) | 0.770 [a] | 25 | 3.8 (2.6, 5.5) | 4 | 2.9 (1.1, 7.2) | 0.608 |
| Prostration | 34 | 2.0 (1.4, 2.8) | 5 | 4.0 (1.7, 9.1) | 0.181 [a] | 13 | 2.0 (1.2, 3.4) | 5 | 3.6 (1.6, 8.2) | 0.219 [a] |
| Other | 29 | 1.7 (1.2, 2.4) | 3 | 2.4 (0.8, 6.9) | 0.473 [a] | 2 | 0.3 (0.1, 1.1) | 1 | 0.7 (0.1, 4) | 0.436 [a] |
| Cyanosis | 17 | 1.0 (0.6, 1.6) | 3 | 2.4 (0.8, 6.9) | 0.148 [a] | 5 | 0.8 (0.3, 1.8) | 1 | 0.7 (0.1, 4) | 1.000 [a] |
| Polypnea | 17 | 1. 0(0.6, 1.6) | 3 | 2.4 (0.8, 6.9) | 0.148 [a] | 5 | 0.8 (0.3, 1.8) | 1 | 0.7 (0.1, 4) | 1.000 [a] |
| Coriza | 12 | 0.7 (0.4, 1.2) | 1 | 0.8 (0.1, 4.4) | 0.598 [a] | 3 | 0.5 (0.2, 1.3) | 1 | 0.7 (0.1, 4) | 0.534 [a] |

[a] Fisher exact test *p*-value.
