# Supplementary material for: Assessment of COVID-19 Vaccine Effectiveness Against SARS-CoV-2 Infection, Hospitalization and Death in Mexican Patients with Metabolic Syndrome from Northeast Mexico: A Multicenter Study
Source: Vaccines (Basel). 2025 Feb 27;13(3):244. doi: 10.3390/vaccines13030244 (PMC11945729; doi:10.3390/vaccines13030244)
Supplement: Supplementary file 1 [file vaccines-13-00244-s001.zip › Table S5.pdf]

Table S5. Symptom presentation in unvaccinated patients and patients vaccinated with two doses of CoronaVac.

	CoronaVac (BO, n = 1,768)					CoronaVac (AO)				
	Unvaccinated (n = 1,714)		Two doses (>14 days) (n = 54)		chi-square p-value	Unvaccinated (n = 658)		Two doses (>14 days) (n = 68)		chi-square p-value
	n	% (95% CI)	n	% (95% CI)		n	% (95% CI)	n	% (95% CI)	
Cough	1,189	69.4 (67.1, 71.5)	37	68.5 (55.3, 79.3)	0.894	519	78.9 (75.6, 81.8)	60	88.2 (78.5, 93.9)	0.067
Headache	1,156	67.4 (65.2, 69.6)	41	75.9 (63.1, 85.4)	0.189	489	74.3 (70.8, 77.5)	46	67.6 (55.8, 77.6)	0.234
Muscle pain	905	52.8 (50.4, 55.2)	25	46.3 (33.7, 59.4)	0.346	328	49.8 (46, 53.7)	27	39.7 (28.9, 51.6)	0.111
Fever	822	48 (45.6, 50.3)	26	48.1 (35.4, 61.1)	0.978	349	53 (49.2, 56.8)	35	51.5 (39.8, 62.9)	0.805
Joint Pain	792	46.2 (43.9, 48.6)	20	37 (25.4, 50.4)	0.183	267	40.6 (36.9, 44.4)	22	32.4 (22.4, 44.2)	0.187
Sore throat	749	43.7 (41.4, 46.1)	32	59.3 (46, 71.3)	0.023	353	53.6 (49.8, 57.4)	38	55.9 (44.1, 67.1)	0.725
Malaise	650	37.9 (35.7, 40.2)	17	31.5 (20.7, 44.7)	0.336	184	28 (24.7, 31.5)	15	22.1 (13.8, 33.3)	0.299
Running nose	586	34.2 (32, 36.5)	20	37 (25.4, 50.4)	0.664	327	49.7 (45.9, 53.5)	39	57.4 (45.5, 68.4)	0.229
Dyspnea	554	32.3 (30.1, 34.6)	10	18.5 (10.4, 30.8)	0.032	93	14.1 (11.7, 17)	4	5.9 (2.3, 14.2)	0.057
Chills	544	31.7 (29.6, 34)	14	25.9 (16.1, 38.9)	0.366	208	31.6 (28.2, 35.3)	30	44.1 (32.9, 55.9)	0.036
Chest pain	359	20.9 (19.1, 22.9)	8	14.8 (7.7, 26.6)	0.274	94	14.3 (11.8, 17.2)	6	8.8 (4.1, 17.9)	0.213
Anosmia	221	12.9 (11.4, 14.6)	7	13 (6.4, 24.4)	0.988	25	3.8 (2.6, 5.5)	6	8.8 (4.1, 17.9)	0.061 [a]
Dysgeusia	204	11.9 (10.5, 13.5)	6	11.1 (5.2, 22.2)	0.860	33	5 (3.6, 7)	4	5.9 (2.3, 14.2)	0.770 [a]
Diarrea	202	11.8 (10.3, 13.4)	8	14.8 (7.7, 26.6)	0.498	38	5.8 (4.2, 7.8)	7	10.3 (5.1, 19.8)	0.179 [a]
Abdominal pain	196	11.4 (10, 13)	8	14.8 (7.7, 26.6)	0.444	66	10 (8, 12.6)	2	2.9 (0.8, 10.1)	0.056
Conjunctivitis	47	2.7 (2.1, 3.6)	2	3.7 (1, 12.5)	0.660 [a]	25	3.8 (2.6, 5.5)	4	5.9 (2.3, 14.2)	0.339 [a]
Prostration	34	2 (1.4, 2.8)	1	1.9 (0.3, 9.8)	1.000 [a]	13	2 (1.2, 3.4)	0	0 (0, 5.3)	0.623 [a]
Other	29	1.7 (1.2, 2.4)	0	0 (0, 6.6)	1.000 [a]	2	0.3 (0.1, 1.1)	1	1.5 (0.3, 7.9)	0.256 [a]
Cyanosis	17	1 (0.6, 1.6)	0	0 (0, 6.6)	1.000 [a]	5	0.8 (0.3, 1.8)	1	1.5 (0.3, 7.9)	0.447 [a]
Polypnea	17	1 (0.6, 1.6)	0	0 (0, 6.6)	1.000 [a]	5	0.8 (0.3, 1.8)	1	1.5 (0.3, 7.9)	0.447 [a]
Coriza	12	0.7 (0.4, 1.2)	0	0 (0, 6.6)	1.000 [a]	3	0.5 (0.2, 1.3)	1	1.5 (0.3, 7.9)	0.326 [a]

[a] Fisher exact test *p*-value.
